# Supplementary material for: Calmodulin as Ca2+-Dependent Interactor of FTO Dioxygenase
Source: Int J Mol Sci. 2021 Oct 8;22(19):10869. doi: 10.3390/ijms221910869 (PMC8509707; doi:10.3390/ijms221910869)
Supplement: Supplementary file 1 [file ijms-22-10869-s001.zip › ijms-1396347-supplementary.pdf]

Supplementary Material

Calmodulin as calcium dependent interactor of FTO dioxygenase

Michał Marcinkowski[1], Tomaš Pilžys[1], Damian Garbicz[1], Jan Piwowarski[1], Kaja Przygońska[1], Maria Winiewska-Szajewska[1], Karolina Ferenc[2], Oleksandr Skorobogatov[1], Jarosław Poznański[1*], Elżbieta Grzesiuk[1*]

[1]Institute of Biochemistry and Biophysics, Polish Academy of Sciences, Pawińskiego 5a, 02-106 Warsaw, Poland

[2]Center of Translational Medicine, Warsaw University of Life Sciences, Nowoursynowska 100, 02-797 Warsaw, Poland

* Corresponding Authors:

Elzbieta Grzesiuk: elag@ibb.waw.pl

Jaroslaw Poznanski: jarek@ibb.waw.pl

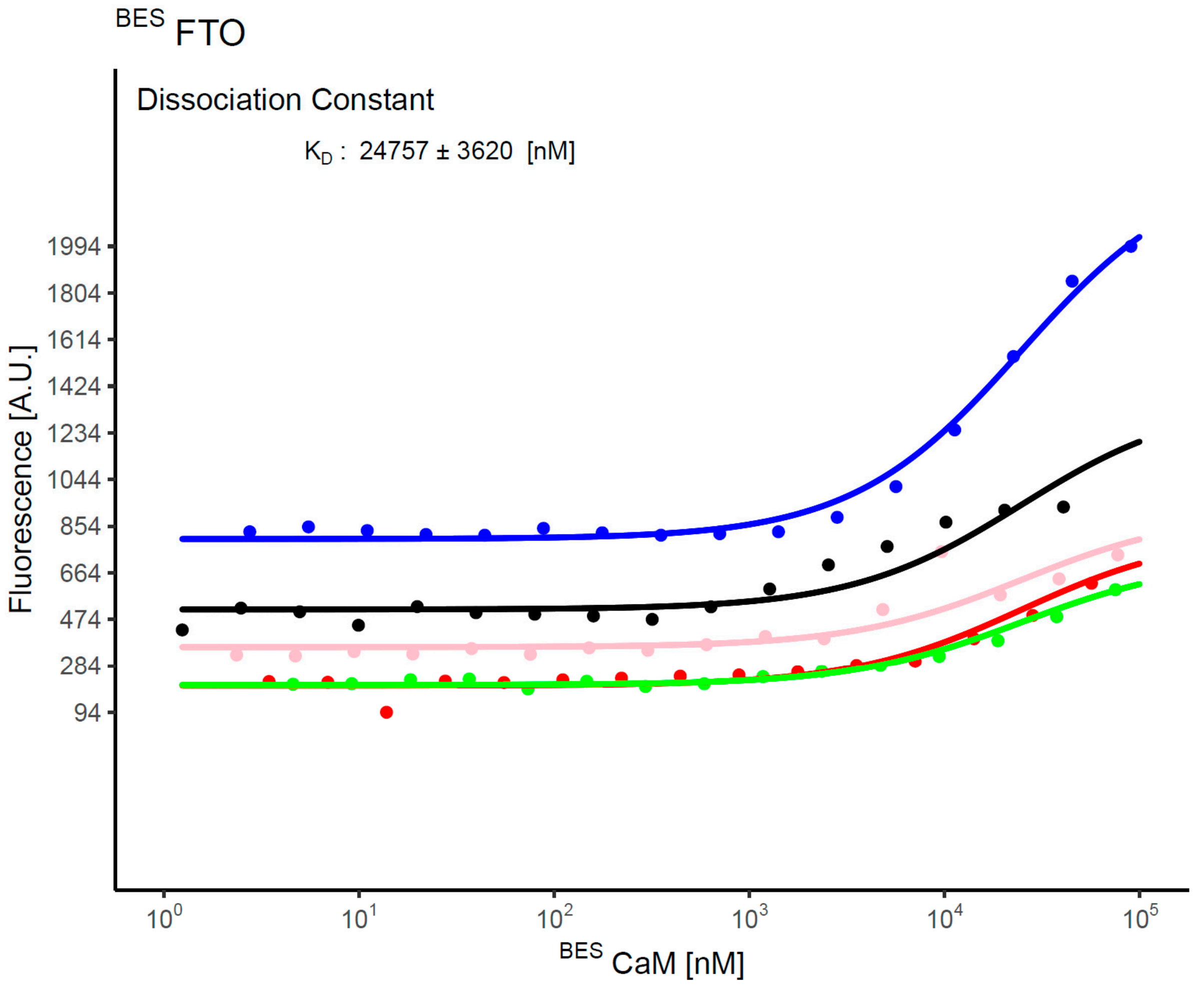

Supplementary Figure S1. Fluorescence analysis of ^{BES}CaM interaction with labeled ^{BES}FTO in the presence of 0.5 mM Fe^{2+}, 0.5 mM Ca^{2+} and 1 mM 2-OG. The image shows that obtained K_D is ~ 1000 times higher with labeled ^{BES}FTO. The plots represents complex level of labeled protein at a given ^{BES}CaM concentration for each separate sample (data points) and for modeled equilibrium between monomer and dimer (straight lines) in each experiment (colors). K_D are shown as estimated value ± standard deviation. Experiment were repeated five times.

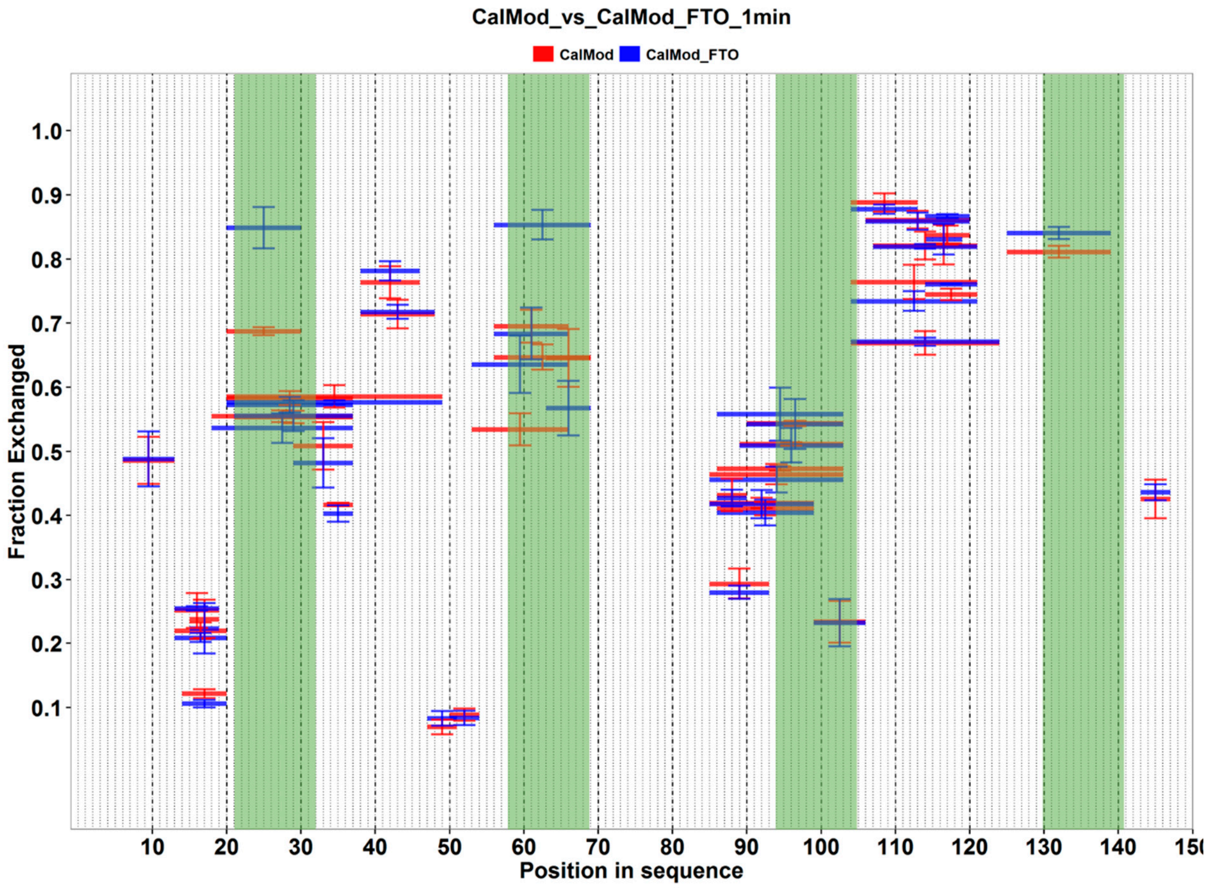

Supplementary Figure S2. HDX profiles of the HDX analysis of the [BES]FTO effect on the solvent accessibility of [BES]CaM after 1 hour incubation with deuterium water. Differences between the exchange levels of individual peptides (each rectangle with whiskers represent one peptide) in samples with and without second protein are shown. Statistically significant differences ($p < 0.05$) in peptide accessibility are marked in purple. Interaction of [BES]CaM with [BES]FTO affects most parts of the first protein, significantly decreasing protein stability after 1 min exchange. In the case of [BES]FTO changes were not so huge, visible after 1 h of exchange, and mostly occurring in C-terminal domain. Each experimental setup was repeated four times.

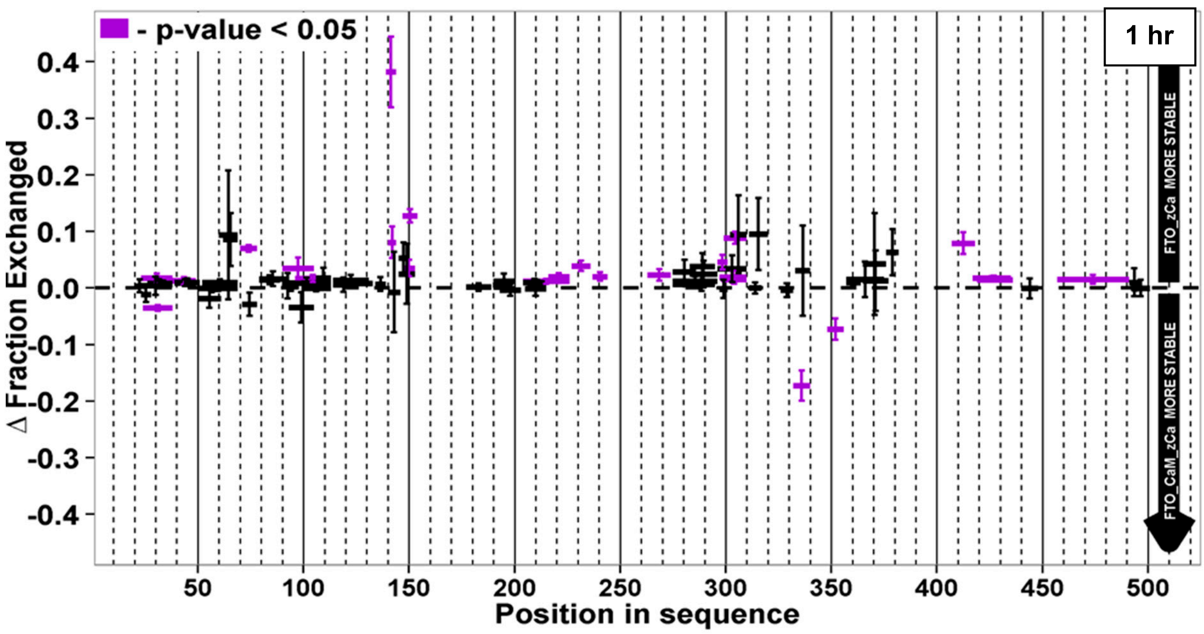

Supplementary Figure S3. HDX analysis of the ^{BES}CaM effect on the solvent accessibility ^{BES}FTO after 1 hour incubation with deuterium water. Differences between the exchange levels of individual peptides (each rectangle with whiskers represent one peptide) in samples with and without second protein are shown. Statistically significant differences ($p < 0.05$) in peptide accessibility are marked in purple. Interaction of ^{BES}CaM with ^{BES}FTO affects most parts of the first protein, significantly decreasing protein stability after 1 min exchange. In the case of ^{BES}FTO changes were not so huge, visible after 1 h of exchange, and mostly occurring in C-terminal domain. Each experimental setup was repeated four times.

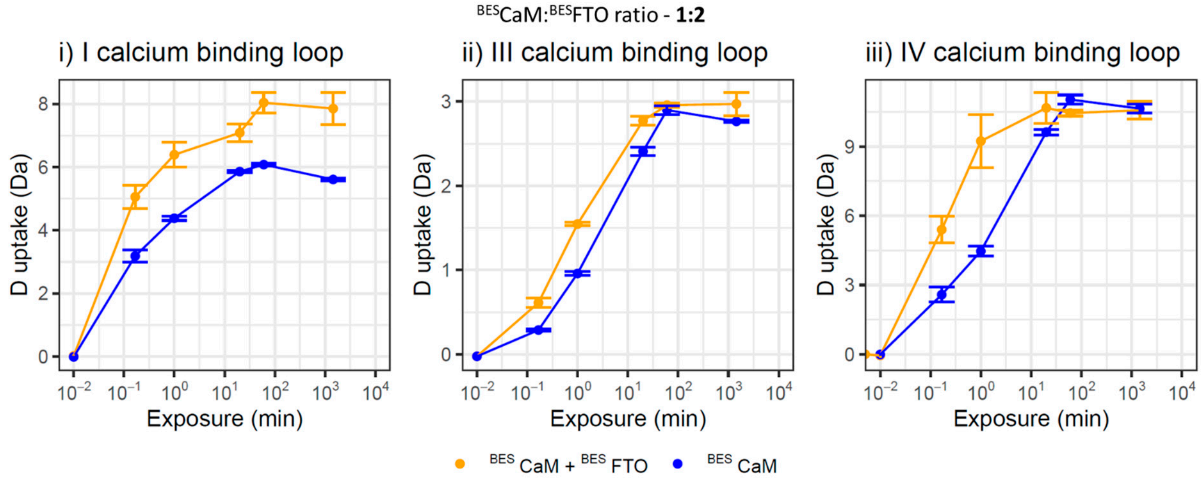

Supplementary Figure S4. Effect of the ^{BES}CaM-^{BES}FTO interaction on the deuterium relative uptake of the selected peptides from ^{BES}CaM calcium binding loops. HDX was monitored in the absence (blue) or presence (orange) of 40μM ^{BES}FTO. Representative deuterium uptake plots for peptide (i) 20-30, (ii) 86-103, (iii) 125-139. Each example shows, that ^{BES}FTO presence increase HD exchange rate of the ^{BES}CaM given peptides. The deuterium incorporation was monitored at 10 sec, 1 min, 20 min, 1 h, and 24 h. Standard deviations for each time point are plotted as error bars. All measurements were performed in quadruplicates.

Supplementary Table S1. PCR primers

FTO C-terminal domain (forward)	5′-TAAGAAGGAGATATACCATGGGCTCAACAGGAACCTTGGATTAT-3′
FTO C-terminal domain (reverse)	5′-GTGGTGGTGGTGGTGCTCGAGGGGTTTTGCTTCCAGAAGCTG-3′
FTO construct (forward)	5′-AGCGGCTCTTCAATGAAGCGCACCCCGACTGCC-3′
FTO construct (reverse)	5′-AGCGGCTCTTCTCCCGGGTTTTGCTTCCAGAAGCT-3′

Amino acid sequences of recombinant FTO proteins. Amino acid sequence of His-Tag is underlined

^{EC}**FTO:**

MGSSHHHHHHSSGLVPRGSHMKRTPTAEEREREAKKLRLLEELEDTWLPYLTPKDDEFYQQWQLK
YPKLILREASSVSEELHKEVQEAFLTLHKHGCLFRDLVRIQGKDLLTPVSRILIGNPGCTYKYLNTRLF
TVPWPVKGSNIKHTEAEIAAACETFLKLNDYLQIETIQALEELAAKEKANEDAVPLCMSADFPRVG
MGSSYNGQDEVDIKSRAAYNVTLLNFMDPQKMPYLKEEPYFGMGKMAVSWHHDENLVDRSAVA
VYSYSCEGPEEESEDDSHLEGRDPDIWHVGFKISWDIETPGLAIPLHQGDCYFMLDDLNATHQHCV
LAGSQPRFSSTHRVAECSTGTLDYILQRCQLALQNVCDDVDNDDVSLKSFEPAVLKQGEEIHNEVEF
EWLRQFWFQGNRYRKCTDWWCQPMAQLEALWKKMEGVTNAVLHEVKREGLPVEQRNEILTAIL
ASLTARQNLRREWHARCQSRIARTLPADQKPECRPYWEKDDASMPLPFDLTDIVSELRGQLLEAKP

^{BES}**FTO:**

MGSSHHHHHHSSGLVPRGSHMKRTPTAEEREREAKKLRLLEELEDTWLPYLTPKDDEFYQQWQLK
YPKLILREASSVSEELHKEVQEAFLTLHKHGCLFRDLVRIQGKDLLTPVSRILIGNPGCTYKYLNTRLF
TVPWPVKGSNIKHTEAEIAAACETFLKLNDYLQIETIQALEELAAKEKANEDAVPLCMSADFPRVG
MGSSYNGQDEVDIKSRAAYNVTLLNFMDPQKMPYLKEEPYFGMGKMAVSWHHDENLVDRSAVA
VYGYSCEGPEEESEDDSHLEGRDPDIWHVGFKISWDIETPGLAIPLHQGDCYFMLDDLNATHQHCV
LAGSQPRFSSTHRVAECSTGTLDYILQRCQLALQNVCDDVDNDDVSLKSFEPAVLKQGEEIHNEVEF
EWLRQFWFQGNRYRKCTDWWCQPMAQLEALWKKMEGVTNAVLHEVKREGLPVEQRNEILTAIL
ASLTARQNLRREWHARCQSRIARTLPADQKPECRPYWEKDDASMPLPFDLTDIVSELRGQLLEAKP

^{EC}**FTO C-terminal domain (327-505)**

MGSTGTLDYILQRCQLALQNVCDDVDNDDVSLKSFEPAVLKQGEEIHNEVEFEWLRQFWFQGNRY
RKCTDWWCQPMAQLEALWKKMEGVTNAVLHEVKREGLPVEQRNEILTAILASLTARQNLRREW
HARCQSRIARTLPADQKPECRPYWEKDDASMPLPFDLTDIVSELRGQLLEAKPLEHHHHHH

^{BES}**CaM:**

MASHHHHHHSGMADQLTEEQIAEFKEAFSLFDKDGDGTITTKELGTVMRSLGQNPTEAELQDMIN

EVDADGNGTIDFPEFLTMVARKMKDTDSEEEIREAFRVFDKDGNGYISAAELRHVMTNLGEKLTDE
EVDEMIREADIDGDGQVNYEEFVQMMTAKGS
